# MicroRNA-331-3p Suppresses Cervical Cancer Cell Proliferation and E6/E7 Expression by Targeting NRP2

**DOI:** 10.3390/ijms17081351

**Published:** 2016-08-18

**Authors:** Tomomi Fujii, Keiji Shimada, Aya Asano, Yoshihiro Tatsumi, Naoko Yamaguchi, Masaharu Yamazaki, Noboru Konishi

**Affiliations:** 1 Department of Pathology, Nara Medical University School of Medicine, Nara 634-8521, Japan; ayaasano1018@yahoo.co.jp (A.A); tatsumi3@naramed-u.ac.jp (Y.T.); nkonishi@naramed-u.ac.jp (N.K.); 2 Department of Diagnostic Pathology, Nara City Hospital, Nara 630-8305, Japan; k-shimada@nara-jadecom.jp; 3 Department of Central Clinical Laboratory, Nara Medical University Hospital, Nara 634-8521, Japan; nyama@naramed-u.ac.jp (N.Y.); masayama@naramed-u.ac.jp (M.Y.)

**Keywords:** cervical cancer, human papillomavirus, miR-331-3p, neuropilin 2, E6/E7 mRNA, keratinocyte differentiation

## Abstract

Aberrant expression of microRNAs (miRNAs) is involved in the development and progression of various types of cancers. In this study, we investigated the role of miR-331-3p in cell proliferation and the expression of keratinocyte differentiation markers of uterine cervical cancer cells. Moreover, we evaluated whether neuropilin 2 (NRP2) are putative target molecules that regulate the human papillomavirus (HPV) related oncoproteins E6 and E7. Cell proliferation in the human cervical cancer cell lines SKG-II, HCS-2, and HeLa was assessed using the 3-(4,5-dimethylthiazol-2-yl)-5-(3-carboxymethoxyphenyl)-2-(4-sulfophenyl)-2*H*-tetrazolium, inner salt (MTS) assay. Cellular apoptosis was measured using the TdT-mediated dUTP nick end labeling (TUNEL) and Annexin V assays. Quantitative RT-PCR was used to measure the messenger RNA (mRNA) expression of the NRP2, E6, E7, p63, and involucrin (IVL) genes. A functional assay for cell growth was performed using cell cycle analyses. Overexpression of miR-331-3p inhibited cell proliferation, and induced G2/M phase arrest and apoptosis in SKG-II, HCS-2 and HeLa cells. The luciferase reporter assay of the NRP2 3′-untranslated region revealed the direct regulation of NRP2 by miR-331-3p. Gene expression analyses using quantitative RT-PCR in SKG-II, HCS-2, and HeLa cells overexpressing miR-331-3p or suppressing NRP2 revealed down-regulation of E6, E7, and p63 mRNA and up-regulation of IVL mRNA. Moreover, miR-331-3p overexpression was suppressed NRP2 expression in protein level. We showed that miR-331-3p and NRP2 were key effectors of cell proliferation by regulating the cell cycle, apoptosis. NRP-2 also regulates the expression of E6/E7 and keratinocyte differentiation markers. Our findings suggest that miR-331-3p has an important role in regulating cervical cancer cell proliferation, and that miR-331-3p may contribute to keratinocyte differentiation through NRP2 suppression. miR-331-3p and NRP2 may contribute to anti-cancer effects.

## 1. Introduction

Cervical cancer is one of the most common neoplasias in females worldwide. The pathogenesis of cervical cancer occurs following persistent infection with high-risk types of human papillomavirus (HPV) such as HPV types 16, 18, 31, 33, 39, 45, 52, 58 and 69; it progresses slowly by interrupting the normal differentiation of the cervical squamous epithelium [1,2]. The E5, E6 and E7 proteins present in the high-risk HPV types, act as viral oncoproteins and are considered to be associated with human cervical carcinogenesis [3,4,5]. E6 and E7 bind to the p53 and retinoblastoma (Rb) family proteins, respectively, which are tumor suppressor proteins involved in cell cycle regulation [5,6]. The inactivation of p53 and Rb proteins by E6 and E7 is likely an important step in cervical carcinogenesis. The recent clinical study suggests that the combination of radiation and molecular targeting therapy with E6/E7 silencing significantly decreased tumor growth in vivo [7].

MicroRNAs (miRNAs) are short regulatory RNAs that control gene expression through translational inhibition by binding to the complementary sites of their target gene transcripts [8,9,10]. miRNAs affect cell proliferation, apoptosis, cell differentiation and the epithelial-mesenchymal transition (EMT) in various types of cancers such as bladder, prostate, breast, pancreatic and gastric cancers [11,12,13,14,15,16]. There are some candidate miRNAs for oncogenic or anti-oncogenic factors in cervical cancer [17]. To evaluate abnormally high-levels of miRNA can potentially serve as useful biomarkers for cervical cancer diagnosis [18]. For example, miR-129-5p induces interferon-β which down-regulates E6 and E7 expression [19], miR-26a and miR-342-3p inhibit cell proliferation and invasion through each protein tyrosine phosphatase type IVA 1 and the mitogen-activated protein kinase (MAPK) pathway or forkhead box M1 [20,21], and miR-101 regulates the cell cycle by inhibiting the G1-to-S transition [22]. miR-196a and miR-181b promote cell proliferation by regulating Forkhead box protein O1 (FOXO1) and p27^Kip1^ or adenylyl cyclase 9, respectively [23,24].

We have previously reported that syndecan-1 (CD138) up-regulates miR-331-3p expression by directly targeting neuropilin 2 (NRP2) and nucleus accumbens-associated protein 1 (NACC1) to mediate the EMT in prostate cancer cells [13]. miR-331-3p inhibits tumor growth, improves prognosis, and regulates the expression of NRP2, deoxyhypusine hydroxylase and the long non-coding RNA Hox transcript antisense intergenic RNA (HOTAIR) and human epithelial growth factor receptor 2 (HER2) in glioblastoma, prostate, and gastric cancers [25,26,27]. In this study, we evaluated whether miR-331-3p regulates cervical cancer proliferation and the expression of the HPV-related proteins E6 and E7 by the candidate targets NRP2. Additionally, to evaluate whether miR-331-3p contributes to keratinocyte differentiation by altering keratinocyte marker expression, we assessed p63 and involucrin (IVL) expression by quantitative reverse transcription-PCR (qRT-PCR).

## 2. Results

### 2.1. miR-331-3p Overexpression Suppresses Cell Proliferation in Cervical Cancer Cells

**Figure 1:**
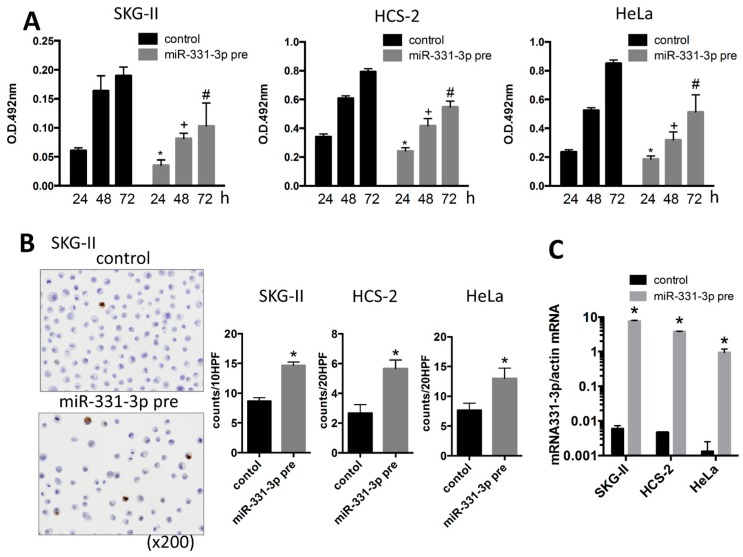
Cell proliferation and apoptosis assay in the cervical cancer cells. (**A**) MTS assay in SKG-II, HCS-2 and HeLa cells. Cell proliferation was suppressed by transient transfection of the miR-331-3p precursor. (pre: precursor, * *p* < 0.05 (24 h), + *p* < 0.05 (48 h), # *p* < 0.05 (72 h)); (**B**) TdT-mediated dUTP nick end labeling (TUNEL) assay for SKG-II, HCS-2 and HeLa cells. The *Y*-axis shows the number of positive cell counts per 10 or 20 high-power fields (10 or 20 HPFs). Positive cells were induced by transfection of the miR-331-3p precursor (* *p* < 0.05); (**C**) Expression of miR-331-3p was up-regulated in SKG-II, HCS-2 and HeLa cells by miR-331-3p precursor transfection compared to control cells (* *p* < 0.05).

**Figure 2:**
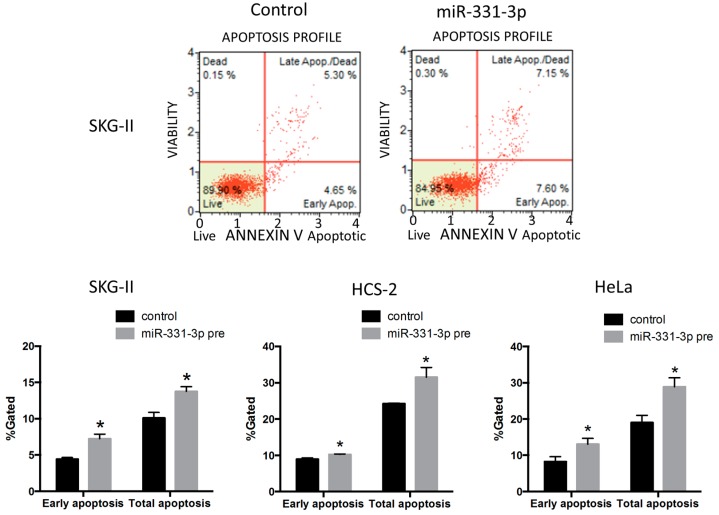
Annexin V assay for cervical cancer cells. Early and total apoptotic cells were significantly increased by miR-331-3p overexpression in SKG-II, HCS-2 and HeLa cells (* *p* < 0.05).

To investigate the roles of miR-331-3p in cervical cancer cells, we performed the MTS assay to evaluate cell proliferation. Overexpression of miR-331-3p significantly decreased cell proliferation in SKG-II, HCS-2 and HeLa cells (Figure 1A). We also assessed the effect of miR-331-3p inhibitor and found that miR-331-3p inhibition did not affect cell proliferation (data not shown). To determine the mechanisms underlying this suppression, we assessed apoptosis by the TdT-mediated dUTP nick end labeling (TUNEL) and Annexin V assays and found that the number of apoptotic cells was significantly increased in SKG-II cells (Figure 1B and Figure 2). The transfection efficiency of miR-331-3p precursor was shown in Figure 1C. The expression level of miR-331-3p was up-regulated 1292-, 820- and 715-fold in SKG-II, HCS-2 and HeLa cells, respectively. We assessed the expression of actin mRNA as the internal control. There was no significant difference in treatment experiments (*C*_t_ value (control/miR-331-3p pre transfected/NRP2 siRNA transfected); SKG-II cells: 13.43 ± 0.08/13.50 ± 0.11/13.55 ± 0.16), HCS-2 cells: 15.17 ± 0.18/15.56 ± 0.10/14.69 ± 0.03, HeLa cells: 14.18 ± 0.17/14.37 ± 0.01/14.28 ± 0.07). Taken together, these data demonstrate that miR-331-3p regulates cell proliferation in cervical cancer cells by inducing apoptosis.

### 2.2. miR-331-3p Significantly Decreases the Expression of HPV-Related Proteins E6 and E7

**Figure 3:**
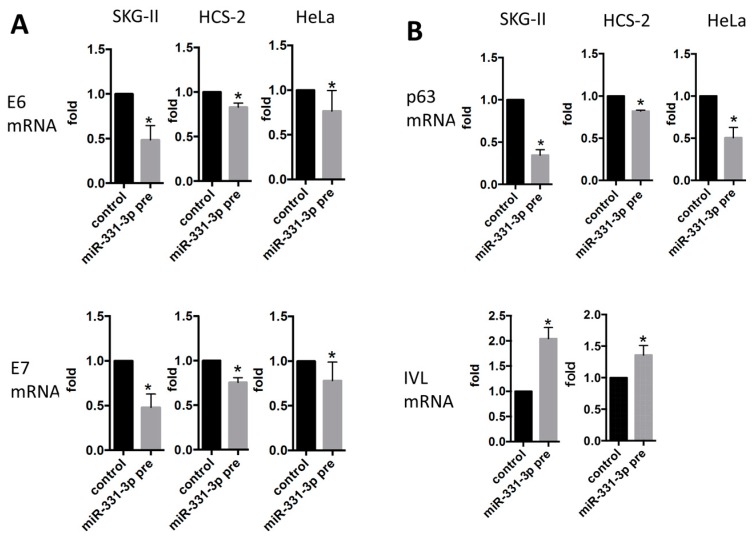
mRNA expression of HPV-related oncogenes, E6/E7 and keratinocyte related genes, p63 and IVL. E6/E7 (**A**) p63 and IVL (**B**) mRNA expression in SKG-II, HCS-2 and HeLa cells. The *Y*-axis (fold) shows relative expression compared with control (normalized with actin mRNA expression). E6/E7 and p63 mRNA expression was significantly decreased in SKG-II, HCS-2 and HeLa cells transiently transfected with miR-331-3p precursor, whereas IVL mRNA was significantly increased in SKG-II, HCS-2 and HeLa cells transiently transfected with the miR-331-3p precursor (* *p* < 0.05).

To assess whether miR-331-3p has functional roles in the regulation of E6 and E7 expression in the HPV-infected squamous cell carcinoma cell lines, we performed qRT-PCR analysis. Overexpression of miR-331-3p significantly suppressed E6 and E7 mRNA expression in SKG-II, HCS-2 and HeLa cells (Figure 3A). miR-331-3p overexpression induced down-regulation of p63 and up-regulation of IVL (Figure 3B); however, suppression of miR-331-3p induced no such changes (data not shown). The data show that miR-331-3p controls expression of E6/E7 and keratinocyte differentiation markers.

### 2.3. Inhibition of Cell Proliferation by miR-331-3p Is Directly Mediated by NRP2 Expression in SKG-II Cells

**Figure 4:**
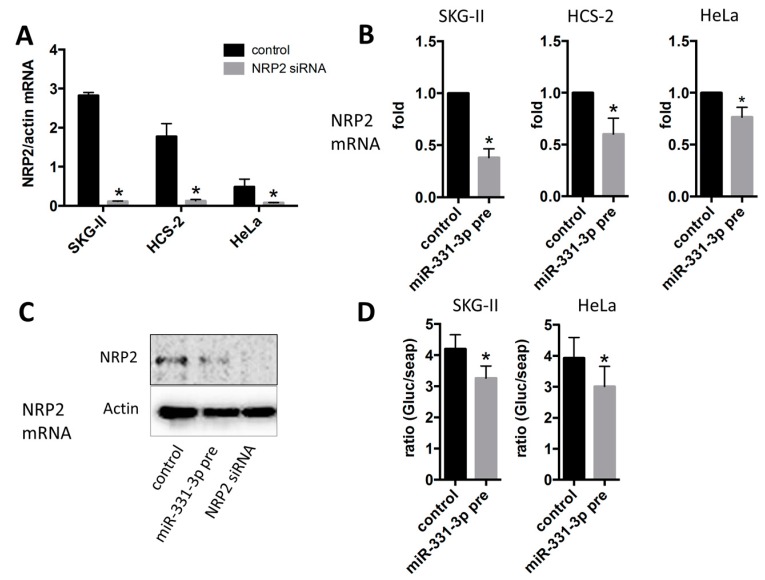
mRNA expression of neuropilin 2 (NRP2), which are the putative target molecules of miR-331-3p. (**A**) mRNA expression of NRP2 in cervical cancer cell lines. NRP2 expression was higher in SKG-II than other two cervical cancer cell lines; (**B**,**C**) NRP2 expression under overexpression of miR-331-3p in cervical cancer cell lines. Images were shown from quantitative RT-PCR (**B**) and western blot (**C**). miR-331-3p down-regulates NRP2 mRNA (**B**) and protein (**C**) expression in SKG-II, HCS-2 and HeLa cells; (**D**) Luciferase reporter activity for NRP-2 3′-UTR. NRP2 3′-UTR reporter activity was reduced by miR-331-3p overexpression (* *p* < 0.05).

**Figure 5:**
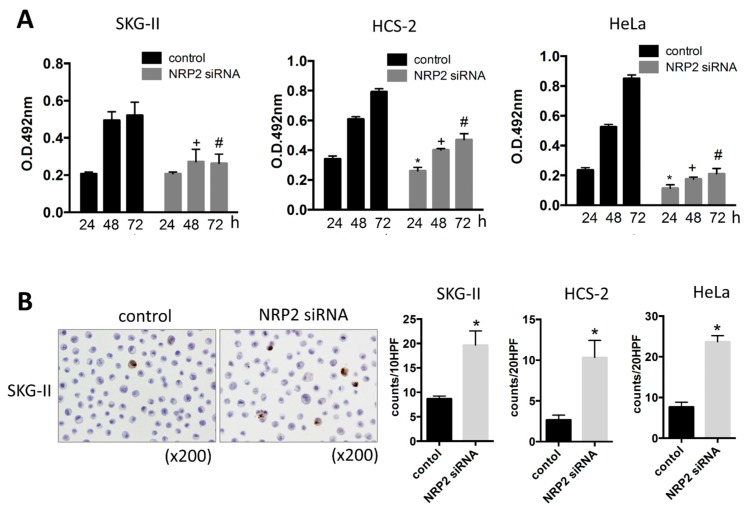
The effect of NRP2 on cell proliferation in SKG-II cells. (**A**) MTS assay in SKG-II, HCS-2 and HeLa cells. Cell proliferation was suppressed by transient transfection with NRP2 siRNA. (* *p* < 0.05 (24 h), + *p* < 0.05 (48 h), # *p* < 0.05 (72 h)); (**B**) TUNEL assay for SKG-II, HCS-2 and HeLa cells. The *Y*-axis shows the number of positive cell counts per 10 or 20 high-power fields (10 or 20 HPFs). Positive cells were induced by transfection of NRP2 siRNA (* *p* < 0.05).

**Figure 6:**
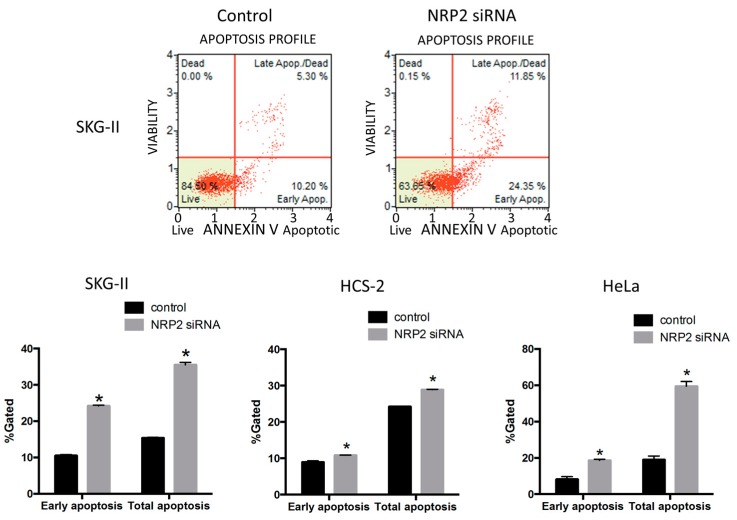
Annexin V assay for cervical cancer cells. Early and total apoptotic cells were significantly increased by NRP2 suppression in SKG-II, HCS-2 and HeLa cells (* *p* < 0.05).

**Figure 7:**
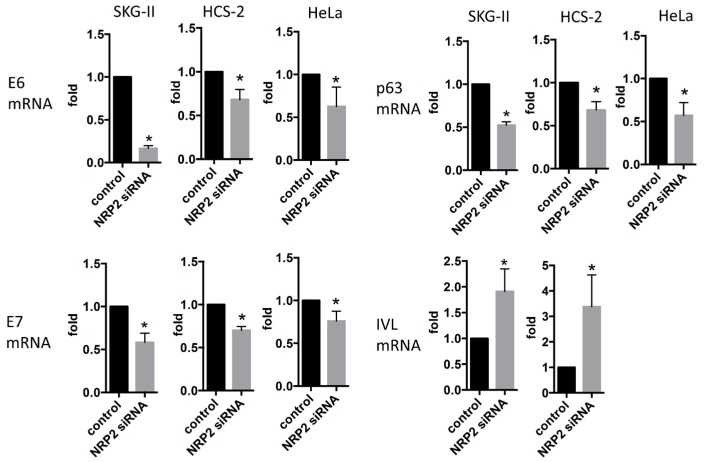
mRNA expression of HPV-related oncogenes, E6/E7 and keratinocyte related genes, p63 and IVL. E6/E7 and p63 mRNAs were significantly decreased by transient transfection with NRP2 siRNA. whereas IVL mRNA was significantly increased in SKG-II, HCS-2 cells transiently transfected with NRP2 siRNA (* *p* < 0.05).

We have previously screened several putative targets of miR-331-3p using the TargetScan analysis (release 6.2, June 2012) and RNAhybrid 2.2 (Bielefeld BioInformatics Service, Bielefeld, Germany) in silico, and identified NRP2 and NACC1 as the predicted targets for miR-331-3p [13]. In this study, we assessed whether these proteins acted as target molecules of miR-331-3p in cervical cancer cells. NRP2 expression was the highest in SKG-II and significantly decreased by miR-331-3p overexpression in SKG-II, HeLa and HCS-2 cells (Figure 4A–C) but NACC1 was not changed by miR-331-3p overexpression in SKG-II cells (data not shown); therefore, NRP2 might act as a target of miR-331-3p in cervical cancer cells. To confirm this, we constructed a Gluc and SEAP reporter cloning vector (pEZX-GA01) and cloned the full-length NRP2 3′-untranslated region (UTR). The effect of miR-331-3p precursor transfection was determined using the luciferase reporter assay (Figure 4D). Suppression of NRP2 expression significantly decreased cell proliferation, whereas the number of apoptotic cells was significantly increased (Figure 5A,B and Figure 6) in SKG-II, HCS-2 and HeLa cells. Moreover, suppression of NRP2 inhibited E6, E7, and p63 expression and induced IVL expression (Figure 7). These results suggest that NRP2 acts directly as a target molecule and is an important for the effect of miR-331-3p on cell proliferation through the expression of E6/E7 and keratinocyte differentiation markers.

### 2.4. Suppression of NRP2 by miR-331-3p Induces G2/M-Phase Cell Cycle Arrest

**Figure 8:**
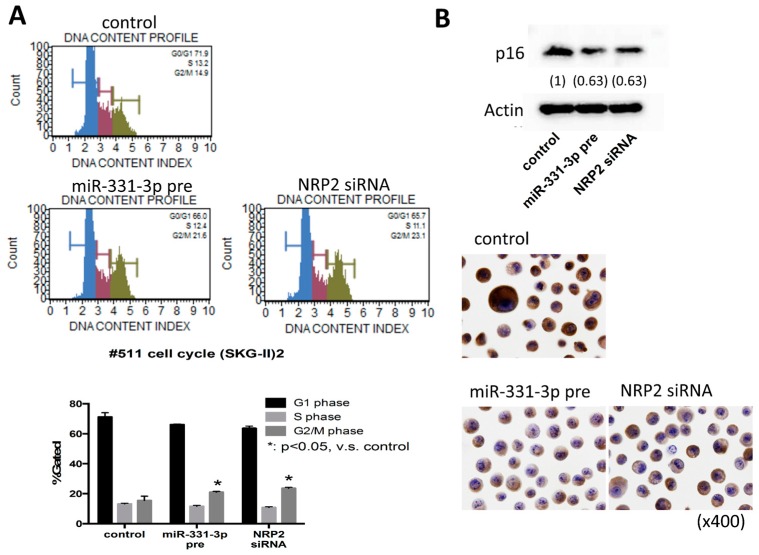
Cell cycle analysis in the SKG-II cells. Cell cycle analysis using the Muse™ Cell Analyzer (Millipore; Hayward, CA, USA). (**A**) The upper panels show the DNA content profile by transient transfection of with the miR-331-3p precursor (miR-331-3p pre) or NRP2 siRNA. The DNA histogram results show the distribution of the cell cycle phases; G0/G1 (blue), S (red) and G2/M (green). The lower panel shows the percentage of G1, S, or G2/M phase; (**B**) Western blotting (**upper panel**) and immunocytochemistory (**lower panel**) for p16INK4a (p16) protein expression in SKG-II cells.

To address the mechanism of the regulation of cell proliferation by miR-331-3p and NRP2, we evaluated the DNA content index. miR-331-3p overexpression or NRP2 suppression increased the number of cells in the G2/M-phase (Figure 8A). To confirm the G2/M-phase arrest in the cell cycle, we performed qRT-PCR using a primer array for cell cycle-related factors. Some molecules related to the G2/M-phase transition were found to be decreased (Figure S1). Western blotting and immunocytochemistry showed that miR-331-3p overexpression and NRP2 inhibition suppressed cytoplasmic p16INK4a protein levels (Figure 8B). These results indicate that miR-331-3p overexpression and NRP2 suppression induce G2/M-phase arrest and down-regulate p16INK4a in cervical cancer cells.

## 3. Discussion

Viral oncogenes derived from HPV play crucial roles in cervical cancer progression. The oncogenic transformation of HPV-infected cancers is triggered by the integration of the viral genome into the host chromosome, which leads to increased E6 and E7 protein expression [28]. In the present study, we showed that miR-331-3p overexpression regulated cell proliferation by inducing cell cycle arrest at the G2/M phase and apoptosis in human cervical cancer cell lines. In addition, NRP2 was found to be a direct target of miR-331-3p, and silencing NRP2 exhibited the same effects on cell proliferation as those observed by miR-331-3p overexpression. Our study clearly suggests that the miR-331-3p and NRP2 axis may play an essential role in the growth of cervical cancer cells.

p63, a member of the p53 family of transcription factors is expressed in basal and parabasal cells of the non-neoplastic squamous epithelium as well as in the neoplastic counterparts including cervical cancer. Immunohistochemical analysis of p63 expression may be an important tool for evaluating most squamous cell carcinomas, such as cervical cancer [29,30]. The life cycle of human HPV is closely related to keratinocyte differentiation. Once the basal cells of squamous epithelium are infected and undergo integration by HPV, the viral genome products E6 and E7 mediate epithelial differentiation [6]. The E6 protein directly affects the IVL promoter to specifically down-regulate IVL expression [31]. In the current study, miR-331-3p overexpression significantly down-regulates NRP2, E6 and E7 proteins, and sequentially up-regulates IVL expression, which may induce keratinocytic differentiation as identified by decreased p63 protein expression in human cervical cancer cells. Taken together, we conclude that miR-331-3p is closely associated with cervical cancer progression by regulating HPV activity.

The cellular protein p16INK4a, which is overexpressed in HPV-infected cervical epithelium, is transformed in response to E7 protein expression [32,33]. p16INK4a is a cyclin-dependent kinase inhibitor that decelerates the cell cycle and functions as a tumor-suppressor gene [34] in many human cancers, in contrast, overexpression of p16INK4a in the nucleus and the cytoplasm strongly correlates with cancer progression in cervical squamous cell carcinomas [35,36]. Therefore, the combined detection of the E6/E7 mRNA and p16INK4a protein is a useful biomarker for histological diagnosis and evaluating of clinical prognosis [34,37,38,39,40,41]. MiR-331-3p has been thought one of cancer-associated miRNAs. In gastric, colorectal and prostate cancers and glioblastoma, miR-331-3p expression was markedly down-regulated [25,27,42,43,44]. In colorectal cancer cells, overexpression of miR-331-3p inhibits cell growth, promotes apoptosis and activates caspase-3 by suppressing HER2 expression [42]. In gastric cancer, miR-331-3p functions as key regulator in cell proliferation by leading to cell cycle arrest through directly suppression of E2F1 gene expression [43]. Therefore, the miR-331-3p could be considered as tumor suppressor genes. On the other hand, miR-331-3p functions as oncogenic miRNA in hepatocellular carcinoma by promoting proliferation and metastasis by directly targeting PH domain and leucine-rich repeat protein phosphatase [45]. Thus, it depends on tumor types whether miR-331-3p acts as tumor-suppressive or oncogenic. Up-to-date, in cervical cancer, there is no report of miR-331-3p and the role of miR-331-3p and NRP2 is still unknown. In our current study, we have shown that miR-331-3p have functioned as the tumor-suppressive miRNA in cervical cancer. NRP2, a putative target of miR-331-3p, which is a co-receptor of semapholin 3 and vascular endothelial growth factor family members, acts as an oncogene and shows high expression in some human carcinomas including lung, colorectal, and pancreatic cancers and glioblastoma [25,46,47,48,49]. In the current study, p16INK4a was significantly decreased with simultaneously suppressed cell proliferation and apoptosis induction through keratinocyte differentiation; decreased p63 and increased IVL expression, by miR-331-3p overexpression or NRP2 suppression were used as indicators of keratinocyte differentiation. These findings suggest that miR-331-3p contributes to the inhibition of progression or to the transformation from high-grade to low-grade through keratinocyte differentiation.

We provide here the key molecules involving the mechanisms by which miR-331-3p overexpression could induce cell cycle arrest at the G2/M phase in human cervical cancer cells using the Primer Array system with emphasis on the E6-p53 and E7-Rb pathways. As a result, Rb mRNA expression was unchanged, and the expression of the p53 and p21 mRNAs was decreased. These results may be mediated by a significant decrease in E6 and E7 following suppression of NRP2 by miR-331-3p, and p53 simultaneously interacts with NRP2 [50], to decrease p21. It is possible that G2/M-phase arrest is caused by the suppression of several G2/M-phase related molecules by miR-331-3p overexpression.

In our current study, we have not shown the clinical significance data in cervical cancer tissues. To offer more evidence of availability as clinical diagnostic tools, it is important to evaluate the miR-331-3p and its target molecules, NRP2 expression using qRT-PCR and immunohistochemistry in cervical cancer and/or dysplasia. In the future, we are going to evaluate expression of miR-331-3p and NRP2 in cervical cancer and dysplasia.

## 4. Materials and Methods

### 4.1. Cell Lines

The human cervical cancer cell lines SKG-II, HeLa and HCS-2 were purchased from the JECB cell bank (National Institutes of Biomedical Innovation, Osaka, Japan). SKG-II cells were cultured in Ham′s F12 media supplemented with 10% fetal bovine serum (NICHIREI BIOSCIENCES INC. Tokyo, Japan) and 50 U/mL penicillin-streptomycin (Nacalai tesque, Kyoto, Japan). The HeLa and HCS-2 cells was cultured in Dulbecco's Modified Eagle Medium (Nacalai tesque) media supplemented with each 10% or 15% fetal bovine serum and 50 U/mL penicillin-streptomycin.

### 4.2. miRNA Precursor and siRNA Transfection in Cervical Cell Lines

For transfection, SKG-II, HeLa and HCS-2 were seeded at each 1.5 × 10^5^ cells/well in a 6-well dish and were transfected with Pre-miR™ miRNA Precursor hsa-miR-331-3p (Life Technologies, Carlsbad, CA, USA) or 100 ng/L siRNA against NRP2 and NACC1 for 72 h. Pre-miR miRNA Precursor Molecules Negative control #2 (Life Technologies) were used as control molecules. Transfection with Pre-miR™ miRNA Precursor or NRP2 siRNA was performed using Lipofectamine RNAiMAX (Life Technologies) in accordance with the manufacturer′s instructions. NRP2 siRNA sequences were designed after selecting appropriate DNA targets as follows: NRP2; 5′-CAGGCTCTGAAGATTGCTCAA-3′.

### 4.3. qRT-PCR Analysis of miRNA and mRNA

For purification of total RNA, including miRNA from cells, we used the miRNeasy Mini kit (QIAGEN, Hilden, Germany). For qRT-PCR, first-strand cDNA was synthesized from 1 µg of total RNA using PrimeScript RT Master Mix (Perfect Real Time) and SYBR Premix Ex Taq II (TliRNaseH Plus) (Takara, Otsu, Japan). The qPCR conditions were 95 °C for 30 s followed by 55–63 °C for 30 s for a total of 35–45 cycles. The primers used were as follows: NRP2 sense 5′-CTGGAAGTCAGCACTAATGGAGAG-3′; NRP2 antisense 5′-GCATCGTTGTTGGCTTGAAATACC-3′; E6 sense 5′-CTCTGTGTATGGAGACACATTGGAA-3′; E6 antisense 5′-GGCACCGCAGGCACCTTA-3′; E7 sense 5′-TAGAAAGCTCAGCAGACGACCTT-3′; E7 antisense 5′-GCACACCACGGACACACAAA-3′; p63 sense 5′-GAAAGCAGCAAGTTTCGGAC-3′; p63 antisense 5′-TTTCATAAGTCTCACGGCCC-3′; IVL sense 5′-AGCCTTACTGTGAGTCTGGTTGA-3′; IVL antisense 5′-GGGTATTGACTGGAGGAGGAACA-3′; Actin sense 5′-CTCTTCCAGCCTTCCTTCCT-3′; Actin antisense 5′-AGCACTGTGTTGGCGTACAG-3′. Gene expression analysis of cell cycle-related genes was performed by qPCR using PrimerArray Cell Cycle (Takara, Otsu, Japan).

### 4.4. Cell Proliferation Assay

The CellTiter 96 AQueous One Solution Cell Proliferation Assay (Promega, Madison, WI, USA) was used for the MTS assay as previously described [51] to measure cell proliferation. Data were collected from triplicate experiments.

### 4.5. Luciferase Reporter Assay

For the luciferase reporter assay, SKG-II and HeLa cells were seeded at 1 × 10^5^ cells per well in a 24-well plate and were transfected after 24 h with 100 ng of *Gaussia* luciferase (Gluc) and secreted alkaline phosphatase (SEAP) reporter plasmid DNA (NRP2 or negative control; Genecopoeia, Rockville, MD, USA) and 5 nM of miR-331-3p precursor. After 24 h, the lysates were assayed for Gluc and SEAP using the Secrete-Pair Dual Luminescence Assay Kit (Genecopoeia, Rockville, MD, USA).

### 4.6. Cell Viability and Cell Cycle Analysis

The cell cycle and Annexin V assays were performed using Muse™ Cell Analyzer from Millipore (Hayward, CA, USA) following the manufacturer’s instructions. Briefly, after transfection of miRNA precursor or siRNA into SKG-II, HCS-2 or HeLa cells, the treated cells were washed with PBS. The cell cycle and Annexin V were then analyzed using the Muse™ Cell Cycle Kit and the Muse^TM^ Annexin V & Dead Cell Assay (Millipore), respectively, according to the manufacturer’s protocol.

### 4.7. Western Blot and Immunocytochemistory Assay

For western blot analysis, the treated cell lysates were separated by Sodium dodecyl sulfate (SDS)-polyacrylamide electrophoresis and transferred onto polyvinylidene difluoride membranes (Millipore), which were then blocked in 5% skim milk at room temperature for 1 h. The membranes were incubated with the indicated primary antibodies, such as anti-NRP2 (Abcam, London, UK) and anti-CDKN2A/p16INK4a (Abcam) for 1 h and then with horseradish peroxidase-conjugated anti-rabbit IgG (Amersham Pharmacia Biotech, Amersham, UK). Peroxidase activity was detected on X-ray films using an enhanced chemiluminescence detection system. For immunocytochemistry, the treated cells were fixed with CytoRich Red Preservative Fluid (BD Biosciences, Franklin Lakes, NJ, USA) and prepared following the SurePath™ method (BD Biosciences). The prepared slides were incubated with the primary antibody (anti-CDKN2A/p16INK4a, Abcam, London, UK) for 1 h at room temperature and the reactions were visualized using a Histofine kit Nichirei, Tokyo, Japan), using diaminobenzidine as the chromogen and hematoxylin as the counterstain.

### 4.8. TdT-Mediated dUTP Nick End Labeling (TUNEL) Assay

SKG-II, HCS-2 and HeLa cells transfected with the miR-331-3p precursor or NRP2 siRNA for 72 h were collected and fixed with CytoRich Red Preservative Fluid (BD Biosciences) and prepared following the SurePath™ method (BD Biosciences). The prepared slides were stained with the ApopTag Plus Peroxidase In Situ Apoptosis Detection Kit (Millipore).

### 4.9. Statistical Analysis

Statistical analysis was performed with GraphPad Prism 6.0 (GraphPad Software, Inc., La Jolla, CA, USA) using a two-tailed Student’s *t*-test to compare between two groups. Graphical data are presented as the mean ± SEM. Results with *p* < 0.05 were considered significant. All experiments were performed in triplicate.

## 5. Conclusions

**Figure 9:**
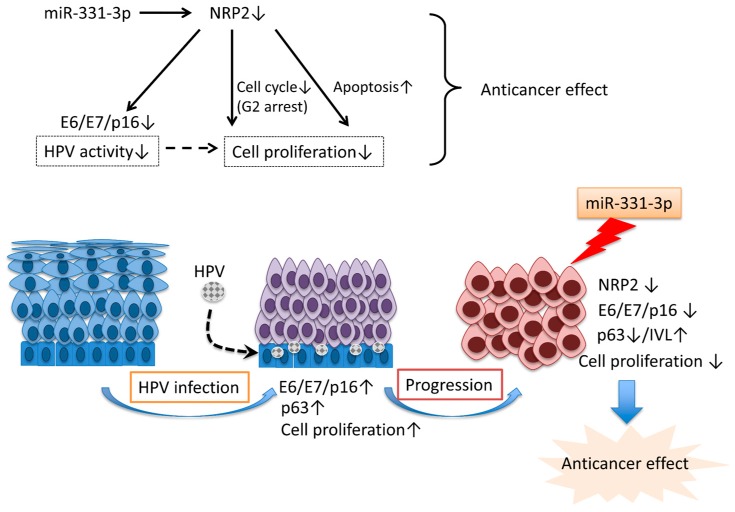
(**Upper panel**) miR-331-3p down-regulates NRP2, a putative direct target of miR-331-3p, and inhibits cell proliferation by regulating the expression of E6/E7, keratinocyte differentiation, cell cycle, and apoptosis in cervical cancer cells (thin black arrows); (**Lower panel**) HPV infection promotes cell proliferation through up-regulation of E6/E7, p16 and p63, and progression to cervical cancer (thin blue arrows). miR-331-3p acts as an anticancer factor through suppression of NRP2 (the thick blue arrow).

In conclusion, these findings show that miR-331-3p overexpression suppresses cervical cancer cell proliferation by regulating the cell cycle and apoptosis (Figure 9). Our future studies will examine whether miR-331-3p and its target, NRP2, are useful clinical diagnostic and/or prognostic markers for histological and cytological examination using tissue specimens and liquid-based cytology in the screening and diagnosis of cervical cancer.

## Supplementary Materials

Supplementary materials can be found at www.mdpi.com/1422-0067/17/8/1351/s1.
