# Supplementary material for: MicroRNA-331-3p Suppresses Cervical Cancer Cell Proliferation and E6/E7 Expression by Targeting NRP2
Source: Int J Mol Sci. 2016 Aug 18;17(8):1351. doi: 10.3390/ijms17081351 (PMC5000747; doi:10.3390/ijms17081351)
Supplement: Supplementary file 1 [file ijms-17-01351-s001.pdf]

Supplementary Material: MicroRNA-331-3p Suppresses Cervical Cancer Cell Proliferation and E6/E7 Expression by Targeting NRP2

Tomomi Fujii, Keiji Shimada, Aya Asano, Yoshihiro Tatsumi, Naoko Yamaguchi, Masaharu Yamazaki and Noboru Konishi

A

symbol	level	symbol	level	symbol	level
TGFB3	0.26	CDC16	0.71	CDKN2D	1.00
CDKN2B	0.30	CDC14A	0.71	GAPDH	1.01
TP53	0.43	HDAC1	0.72	E2F3	1.01
YWHAG	0.47	MAD2L2	0.73	HPRT1	1.04
CREBBP	0.49	CUL1	0.73	SKP1A	1.04
ORC5L	0.50	B2M	0.73	CDK6	1.06
CDC25B	0.51	CCNB2	0.74	RB1	1.06
CDKN2C	0.51	TBP	0.74	CDC23	1.10
ABL1	0.53	PPIA	0.74	RBX1	1.13
SMAD4	0.54	YWHAE	0.76	SMAD3	1.18
MCM5	0.55	ORC3L	0.76	FZR1	1.20
WEE1	0.55	CDC7	0.76	PGK1	1.30
GADD45A	0.55	CDC2	0.77	SMC1A	1.43
CDC6	0.55	YWHAQ	0.78	TGFB1	1.46
CDC25A	0.56	E2F4	0.78	CCND1	1.65
MCM7	0.57	BUB1B	0.82		
MAD1L1	0.59	CDK7	0.82		
MDM2	0.60	CDC27	0.83		
GSK3B	0.61	MAD2L1	0.84		
BUB3	0.61	ATR	0.86		
GADD45B	0.61	RBL1	0.86		
ANAPC5	0.62	CCNB1	0.86		
ANAPC4	0.63	CDC14B	0.88		
CHEK1	0.63	TGFB2	0.88		
E2F1	0.64	TFDP1	0.89		
MCM4	0.64	BUB1	0.90		
GUSB	0.65	PCNA	0.91		
SMAD2	0.65	YWHAB	0.91		
PLK1	0.66	RBL2	0.92		
CHEK2	0.66	SKP2	0.93		
DBF4	0.66	HDAC2	0.93		
ANAPC10	0.66	PRKDC	0.94		
SFN	0.66	CCNA1	0.94		
CDC45L	0.66	YWHAZ	0.95		
ORC1L	0.67	CCND3	0.95		
ORC4L	0.68	CDKN1A	0.95		
CDK2	0.69	E2F5	0.96		
CCNE1	0.69	ATM	0.97		
CCNA2	0.69	CDC20	0.99		
MCM3	0.70	ACTB	0.99		

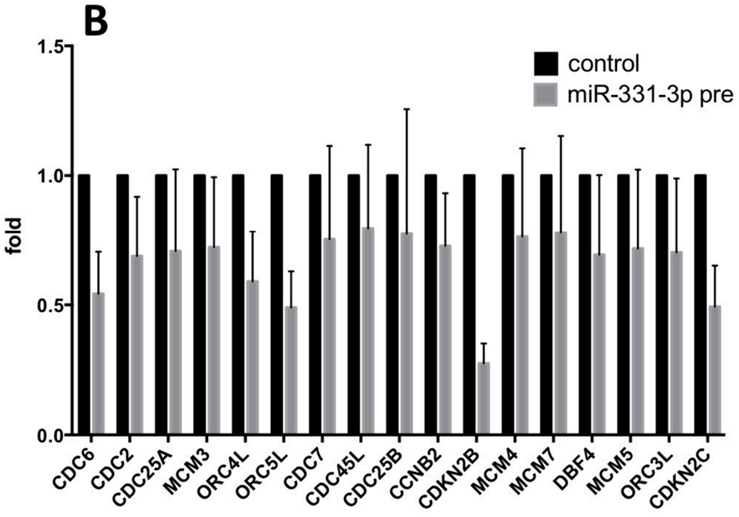

Figure S1. Gene expression analysis using the PrimerArray® Cell Cycle (Human). (**A**) The PrimerArray® Analysis Tool Ver.2.2 (TaKaRa, Otsu, Japan) was used for RT-PCR data analysis tool. The genes indicated in blue were significantly decreased by less than 0.50. Genes indicated in green were slightly decreased by less than 0.70. There is no genes that significantly increased by more than 2.00; (**B**) Some G2/M-phase related genes were decreased by transfection with the miR-331-3p precursor in SKG-II cells.
